# Prefrontal Response and Frontostriatal Functional Connectivity to Monetary Reward in Abstinent Alcohol-Dependent Young Adults

**DOI:** 10.1371/journal.pone.0094640

**Published:** 2014-05-07

**Authors:** Erika E. Forbes, Eric E. Rodriguez, Samuel Musselman, Rajesh Narendran

**Affiliations:** 1 Department of Psychiatry, University of Pittsburgh School of Medicine, Pittsburgh, Pennsylvania, United States of America; 2 Department of Psychology, University of Pittsburgh, Pittsburgh, Pennsylvania, United States of America; 3 Department of Pediatrics, University of Pittsburgh School of Medicine, Pittsburgh, Pennsylvania, United States of America; 4 Department of Radiology, University of Pittsburgh School of Medicine, Pittsburgh, Pennsylvania, United States of America; Erasmus University Rotterdam, Netherlands

## Abstract

Although altered function in neural reward circuitry is widely proposed in models of addiction, more recent conceptual views have emphasized the role of disrupted response in prefrontal regions. Changes in regions such as the orbitofrontal cortex, medial prefrontal cortex, and dorsolateral prefrontal cortex are postulated to contribute to the compulsivity, impulsivity, and altered executive function that are central to addiction. In addition, few studies have examined function in these regions during young adulthood, when exposure is less chronic than in typical samples of alcohol-dependent adults. To address these issues, we examined neural response and functional connectivity during monetary reward in 24 adults with alcohol dependence and 24 psychiatrically healthy adults. Adults with alcohol dependence exhibited less response to the receipt of monetary reward in a set of prefrontal regions including the medial prefrontal cortex, lateral orbitofrontal cortex, and dorsolateral prefrontal cortex. Adults with alcohol dependence also exhibited greater negative correlation between function in each of these regions and that in the nucleus accumbens. Within the alcohol-dependent group, those with family history of alcohol dependence exhibited lower mPFC response, and those with more frequent drinking exhibited greater negative functional connectivity between the mPFC and the nucleus accumbens. These findings indicate that alcohol dependence is associated with less engagement of prefrontal cortical regions, suggesting weak or disrupted regulation of ventral striatal response. This pattern of prefrontal response and frontostriatal connectivity has consequences for the behavior patterns typical of addiction. Furthermore, brain-behavior findings indicate that the potential mechanisms of disruption in frontostriatal circuitry in alcohol dependence include family liability to alcohol use problems and more frequent use of alcohol. In all, these findings build on the extant literature on reward-circuit function in addiction and suggest mechanisms for disrupted function in alcohol dependence.

## Introduction

Across conceptual models, disrupted function in neural reward circuitry is postulated to be a central mechanism of alcohol dependence and other forms of addiction [1], [2], [3]. Changes in reward circuitry with the development of addiction are thought to reflect a shift from reward-driven behavior during the initial stages of addiction to loss of reward function by later stages [4]. Recent models of addiction have focused on altered function in prefrontal regions that mediate behavioral processes such as compulsivity, impulsivity, and higher-order cognitive control [5], [6]. Together, these constructs reflect difficulty in controlling behavior flexibly, as directed by future goals rather than short-term gains, and through inhibition of inappropriate or goal-inconsistent behaviors, respectively. Specifically, adaptations related to the development of addiction are posited to involve increased hypofrontality, which can be conceptualized as poor executive control over behavior, as well as poor modulation of responding in other reward regions. In terms of prefrontal function, these adaptations are likely to involve altered function in regions such as the orbitofrontal cortex (OFC), medial prefrontal cortex (mPFC), and dorsolateral prefrontal cortex (DLPFC), all of which contribute to function in reward circuitry but have differing roles. For example, although all of these regions process rewarding stimuli, the mPFC can have an excitatory influence on dopamine neurons [7] and seems to respond to contextual features of reward [8], while the OFC appears to specialize in inhibition of dopamine neurons [7]. Furthermore, subregions of the OFC differ: medial OFC appears specialized for responding to reward and lateral OFC for responding to punishment and to changing or suppressing previously rewarded behaviors [9], [10]. In addition, Both imaging and basic science studies indicate that chronic drug or alcohol use leads to a disruption in the OFC regulation of ventral striatum (primarily, nucleus accumbens) DA transmission via glutamatergic projections (for a review, see [11]).

In addition, adaptations in dopamine-system function are postulated to disrupt the functional connectivity of these prefrontal regions with the ventral striatum (VS), especially the nucleus accumbens. For example, in a positron emission tomography study, Volkow and colleagues reported that controls—but not alcohol-dependent adults—exhibited an association between decreased glucose metabolism in the OFC and increased methylphenidate-induced dopamine release in the VS [12]. This finding suggests altered coupling between these two regions, with an association between lower response in the OFC and increased dopamine in the VS, which could indicate less effective regulation of VS responding by OFC. Furthermore, in detoxified alcohol-dependent adults, weaker OFC functional connectivity with the midbrain predicts the likelihood of relapse [13]. Similarly, this finding raises the possibility that poor or suboptimal connectivity between OFC and basic reward regions is a correlate or consequence of addiction. Given the role of the OFC in inhibitory control and self-regulation [14], [15], these findings suggest that alcohol dependence involves reduced inhibition of ventral striatal response, which is relevant to compulsive use.

Non-drug rewards such as money or natural rewards can provide a valuable context for investigating hypofrontality in alcohol dependence. For two possible reasons, responding in frontostriatal circuitry is likely disrupted during the processing of non-drug rewards. First, lasting, generalized hypofrontality in people suffering from addiction could be evident in neural response to a variety of reward classes, including to non-drug rewards such as money or natural rewards such as food. Changes in the dopamine system influence response to both drug rewards (i.e., the drug itself, with its rewarding effects) and non-drug rewards [15], and it is likely that the neural adaptations that occur with addiction generalize to responding in the presence of other types of reward, including natural rewards and money. Thus, hypofrontality could reflect general disruption in reward circuitry, whether responding to drug or non-drug rewards. In the case of non-drug rewards, hypofrontality could have consequences for difficulty in guiding behavior to obtain these rewards as necessary for healthy functioning [11]. Second, another possibility is that hypofrontality is particularly evident in the context of response to non-drug or natural rewards in addiction, because reward circuitry adapts to focus on drug rewards at the expense of non-drug rewards. Consistent with this possibility, studies of alcohol dependence have revealed an opposite pattern of response to alcohol-related and non-alcohol-related stimuli in addiction. Specifically, it appears that in comparison with healthy adults, alcohol-dependent adults exhibit greater response in reward-related regions to alcohol-related reward [16], [17], [18], [19], but *decreased* response to monetary (i.e., non-drug) reward [20], [21]. Furthermore, response in the mPFC and VS to alcohol-related reward predict relapse among detoxified alcohol-dependent adults [22]. Through either process, reduced coupling of prefrontal cortical regions from the ventral striatum has been hypothesized to diminish and enhance the capacity of the prefrontal cortex to initiate behaviors in response to natural and alcohol-related reward stimuli, respectively, which in turn drives the cycle of addiction [11].

Developmentally, it is critical to examine alterations in PFC response to reward in alcohol dependence early in adulthood. Not only is this period early in the course of dependence, which provides greater opportunity to examine the development of addiction and less severity of the toxic effects of alcohol exposure [23], [24], but early adulthood is a window in which dopamine is exerting a decreasing influence on PFC function [25]. Low VS reponse to monetary reward has been reported in children of alcohol-dependent parents, indicating that this characteristic may be a risk factor for the development of alcohol-related problems [26]. In the PFC, it is especially important to investigate this issue during early adulthood because dopamine influence on PFC function changes across adult development, resulting in changes in function of reward circuitry as well as altered associations between dopamine synthesis and PFC function [25]. Specifically, with adult development, dopamine synthesis decreases in cortex but increases in dorsal striatum, and the shift toward greater dopamine synthesis in the dorsal caudate is associated with lower performance on executive function tasks [27]. These findings suggest that assessing dopamine-related functioning in PFC—for example, during reward processing—is more challenging in older adults, who have experienced declines in PFC dopamine functioning. Thus, to understand dopamine-mediated alterations in PFC control of reward responding, it is optimal to investigate PFC function at a developmental period proximal to development of the dopamine system. Alcohol-dependent young adults in their 20 s provide a valuable group for addressing this research question. Although much of their PFC maturation is completed, their PFC dopamine signaling has not yet declined, they are likely to be early in the clinical course of alcohol dependence, and they are relatively young for people in the alcohol-dependent population.

We examined neural response to monetary reward in 24 young adults with alcohol dependence and 24 healthy young adults. Using a reliable fMRI task involving guessing numbers and winning money, we predicted that alcohol dependence would be related to hypofrontality, defined as low response in OFC, mPFC, and DLPFC. We also predicted that alcohol dependence would be associated with altered frontostriatal functional connectivity, defined as greater negative correlation in task-related functional connectivity between those PFC regions and the nucleus accumbens. Because we focused on monetary reward, we predicted that alcohol dependence would be associated with low VS response. Finally, to address the possible mechanisms leading to group differences, we examined brain-behavior associations involving drinking characteristics such as frequency and quantity of drinking, number of years drinking, and family history of alcohol dependence. Examining associations of these characteristics with neural response and functional connectivity within the alcohol dependent group provided the opportunity to obtain a more detailed understanding of the process by which frontostriatal function could develop from patterns of drinking behavior or genetic vulnerability (in the case of family history).

## Materials and Methods

### Ethics Statement

Procedures were approved by the University of Pittsburgh Institutional Review Board. All participants provided written informed consent to study procedures.

### Participants

Forty-eight young adults (24 with alcohol dependence, 24 healthy controls) underwent fMRI in a Siemens 3 T Trio scanner during a block-design monetary reward task. The groups were matched for age, sex, race, and smoking habits. Sample characteristics are presented in Table 1. As expected, the alcohol dependent group did not differ from the healthy control group in any demographic characteristics. Participants were recruited in the greater Pittsburgh area from a larger PET study and from community advertisements. The original total sample included 59 individuals. Of these, 9 were unable to complete the functional tasks in the fMRI scanner either due to scheduling limitations or scanner malfunction, 2 individuals were excluded for low-response rates in the scanner (less than 50% of total responses), and 1 was excluded for inadequate coverage of prefrontal regions. Exclusion criteria for this study included the following: pregnancy, serious medical or neurological illness, systolic blood pressure >140 mmHG, diastolic blood pressure <90 mmHG, any current major axis I psychiatric diagnosis (except alcohol and nicotine use disorder), metal implants or paramagnetic objects, employment as a radiation worker or exposure to radiation that exceeds annual dose of radiation, medical history of chronic obstructive pulmonary disease or other chronic respiratory disorders, and renal or liver problems.

**Table 1:** Sample Characteristics.

	Alcohol Dependent Group	Healthy Control Group	Group Differences
N	24	24	
Age	27.2±4.9	27.2±3.7	*F*(1,46) = .00, *ns*
Sex	37.5% Female	41.7% Female	?^2^ = .09, *ns*
Race	79.2% Caucasian	79.2% Caucasian	?^2^ = .00, *ns*
Education: Some College	58.3%	83.3%	?^2^ = 4.04, *ns*
Daily Smokers (*n*)	12	12	?^2^ = .00, *ns*
Task Reaction Time (ms)	805.0±161.7	843.4±213.8	*F*(1,46) = .49, *ns*
Frequency of drinking (days/week)	5.83±1.59	N/A	
Severity of Alcohol Dependence	20.21±5.53	N/A	
Drinks/Use	11.61±5.53	N/A	
Years Drinking	10.46±5.69	N/A	
Family History of Alcohol Dependence	79.2%	N/A	
Days since Last Use	48.33±60.54	N/A	

*Note*: All participants in the alcohol dependent group met DSM-IV criteria for alcohol dependence but had undergone monitored abstinence prior to participating. Severity of alcohol dependence was measured with the Alcohol Dependence Scale [62]. All daily smokers met criteria for nicotine dependence. Family history reflects alcoholism in any first- or second-degree relatives. Groups did not differ significantly for any demographic characteristics. N/A: other than confirming the absence of alcohol use disorders, we did not assess drinking behavior and drinking history in the healthy control group.

Lifetime psychiatric diagnoses in all participants were assessed using the Structured Clinical Interview for DSM-IV [28]. Participants in the alcohol group had current DSM-IV-defined Alcohol Dependence and had undergone a 2-week monitored abstinence. Outpatient monitored abstinence from alcohol and other recreational drugs were confirmed with ethyl glucuronide/ethylsulfate (ETG/ETS) and urine drug screens performed three times/week for two consecutive weeks before assessment. During this monitoring period, participants were paid $75 for each negative ETG/ETS urine to promote abstinence from alcohol. All alcohol dependent subjects were monitored for alcohol withdrawal during the first week of abstinence using the Clinical Institute Withdrawal Assessment of Alcohol Scale (CIWA-Ar). Individuals who scored greater than 19 on the CIWA-Ar during the initial intake evaluation (*n* = 0 for the current study) and/or with a prior history of alcohol withdrawal seizures or delirium tremens were excluded from the research protocol. Participants in the comparison group were free of lifetime psychiatric disorders. All participants were in good medical health and underwent a physical exam and bloodwork prior to entering the study.

### Response of Neural Reward Circuitry

#### Paradigm

**Figure 1:**
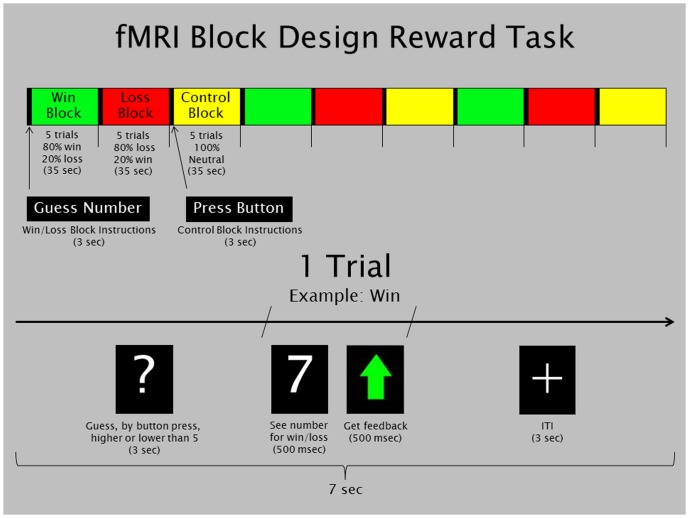
The block-design monetary reward task employed in the current study. Participants were instructed to guess whether a number was low or high and to respond to win outcomes.

The fMRI paradigm was a slow event-related card-guessing game [29] that allows examination of response to monetary reward and reliably engages the striatum (see Figure 1). Participants received win, loss, or a neutral control feedback for each trial. Participants were told that their performance would determine a post-scan monetary reward, with $1 for each win and 50 cents deducted for each loss with no money being gained or deducted for the control blocks. The 45 trials were divided into three different block types: win, loss, and control. These blocks were presented in fixed, pseudorandom order with predetermined outcomes that were identical across participants. During each win and loss trial, participants guessed via button press whether the value of a hidden number was high or low (3 s); learned the value of the hidden number (.5 s); and received outcome feedback (.5 s). Feedback consisted of a green upward-facing arrow for a win outcome and a red downward-facing arrow for a loss outcome. A crosshair was then presented for 3 s (intertrial interval), for a total trial length of 7 s. The motor control aspect of this task operated in the same fashion as the win/loss trials. Participants were asked to press a button when presented with an “X” (3 s), which was followed by an asterisk (.5 s), and were presented with a yellow circle for the neutral outcome (.5 s). The contrast of interest derived from the task for all analyses was *win > loss*. The control condition was not included in analyses.

Participants were unaware of fixed outcome probabilities, and their engagement and motivation were maintained by verbal encouragement between runs. Participants practiced the task before the scan and did not exhibit a change in reaction time across task runs during the scan. The alcohol group and comparison group did not differ in mean reaction time during the task.

### fMRI acquisition, processing, and analysis

Each participant was scanned using a Siemens Trio 3 T scanner. BOLD functional images were acquired with a gradient echo planar imaging (EPI) sequence and covered 34 axial slices (3 mm thick) beginning at the cerebral vertex and encompassing the entire cerebrum and the majority of the cerebellum (TR/TE = 2000/29 ms, FOV = 200×200, matrix = 64×64). All scanning parameters were selected to optimize the quality of the BOLD signal while maintaining a sufficient number of slices to acquire whole-brain data. Before the collection of fMRI data for each participant, we acquired a reference EPI scan that we visually inspected for artifacts (e.g., ghosting) and for good signal across the entire volume of acquisition. The fMRI data from all included participants were cleared of such problems.

Whole-brain image analysis was conducted using SPM8 (http://www.fil.ion.ucl.ac.uk/spm). For each scan, images for each participant were realigned to correct for head motion. Data sets were then selected for quality based on our standard small-motion correction (<2 mm) and for signal coverage in the striatal region of interest >80%. Realigned images were co-registered to structural images that had been segmented to select grey matter, then spatially normalized into standard stereotactic space (Montreal Neurological Institute template) using a 12-parameter affine model. Normalized images were smoothed with a 6 mm full-width at half-maximum Gaussian filter. Voxel-wise signal intensities were ratio normalized to the whole-brain global mean.

Preprocessed data sets were then analyzed using first-level random effects models that account for scan-to-scan variability and second-level random effects models that account for participant-to-participant variability to determine task-specific regional responses. For each participant and scan, condition effects (i.e., main effects of task) at each voxel were calculated using a t-statistic, producing a statistical image for the contrast of interest (i.e., win > loss).

### Data Analyses

We examined neural response to win vs. loss in the following five *a priori* neural regions of interest (ROIs): (1) ventral and dorsal striatum; (2) mPFC, including medial BA9, medial BA10, and BA32 (24); (3) medial OFC, defined as BAs 11 and 12; (4) lateral OFC, defined as BA47; and (5) DLPFC. We distinguished medial from lateral OFC because of claims that these two subregions differ in function, with medial OFC specializing in value-related choice and lateral OFC specializing in reward learning, inhibitory control, and regulation of reward responding [30], [31]. In addition, DLPFC was included because of emerging findings indicating its disruption in addiction, especially during decision-making [32]. ROIs were defined anatomically using the WFU PickAtlas Tool (v3.0.4), either by creating a sphere around a set of central coordinates (for VS, 10 mm radius around [0, 10, −10], [33]; for mPFC, 20 mm radius around [0, 42, 18], [34]) or by selecting a region (i.e., BA47 for lateral OFC; BAs 11 and 12 for medial OFC; BAs 9 and 46 for DLPFC). The mPFC ROI was designed to focus on dorsal mPFC, so that it would not overlap with the medial OFC ROI.

We tested group differences in neural response using second-level random-effects factorial models in SPM. We examined functional connectivity using interaction (PPI) analyses in SPM8, with the bilateral nucleus accumbens, defined by the PickAtlas anatomical region, as the seed region. The psychological variable for PPI was win > loss, which serves to isolate response to reward from response to feedback generally. Because the contrast for PPI involved blocks for which BOLD response would not overlap, our task timing was appropriate for PPI analyses. Sex, race, and daily smoking quantity were included as covariates in analyses for group differences. We investigated brain-behavior analyses focusing on drinking behavior by conducting regressions in SPM within the alcohol dependent group. These regression analyses included age as a covariate. There were no evident sex differences in drinking behaviors, and accordingly we did not include sex as a covariate in these regression analyses. All analyses initially used a voxel-wise threshold of *p*<.05 and a minimum extent of 10 contiguous voxels. We then used simulations in AlphaSim based on the five-ROI mask to compute minimum cluster sizes in order to adjust for Type I error in all analyses. The minimum extent size of continuous voxels required for a corrected *p*<.05 cutoff using the entire mask was 247 voxels.

## Results

### Behavior

Participants responded to a high proportion of trials (97.7%±10.0%). As expected, the alcohol dependent and control groups did not differ in reaction time or proportion of trials with responses during the task (Table 1).

### Group Differences in Neural Response to Monetary Reward

**Figure 2:**
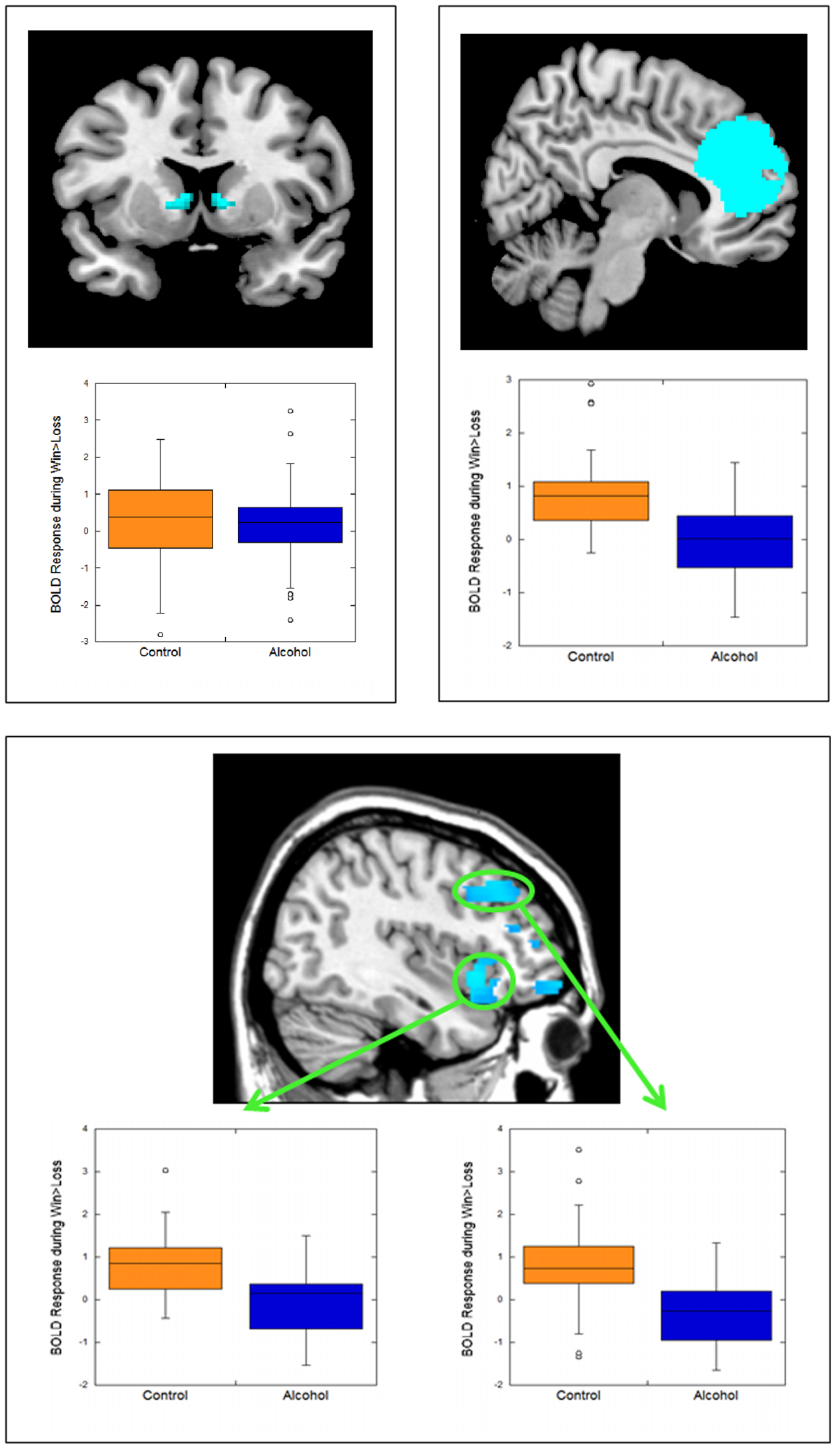
Alcohol dependent adults exhibited less response to monetary win vs. loss than healthy adults in three prefrontal regions—the medial prefrontal cortex (top right panel), the lateral orbitofrontal cortex (bottom panel, left boxplot), and the dorsolateral prefrontal cortex (bottom panel, right boxplot)—as well as the ventral striatum (top left). Boxplots illustrate findings by depicting mean BOLD response across the entire indicated functional cluster for each region, by group.

The alcohol dependent group showed less response in the lateral OFC, mPFC, DLPFC, and VS (anteroventral caudate region, extending into dorsal caudate) than the comparison group to win vs. loss (Table 2; Figure 2). The alcohol dependent group did not exhibit greater response than the comparison group in any of the ROIs. The groups did not differ in medial OFC response. There were no sex differences in neural response. Whole-brain results for group differences confirmed that these ROIs were critical regions in which the groups differed (Table S1).

**Table 2:** Differences in Function of Reward Circuitry between Adults with Alcohol Dependence and Healthy Control Adults.

Regions	BA	Cluster Size	*t*-score at peak voxel	Talairach coordinates of peak voxel		
				x	y	z
***Alcohol < Control, Neural Response***						
Right Lateral OFC	47	780	5.69	32	12	1
mPFC, Right DLPFC	32,8	5782	5.49	2	27	32
Right Lateral OFC	10,11	537	4.89	33	53	1
Left Lateral OFC	13	454	4.86	−36	12	−1
Ventral Striatum, Dorsal Striatum		255	3.88	−10	2	8
Left Lateral OFC	10,11	328	3.99	−23	50	−6
***Alcohol > Control, Negative Functional Connectivity with Bilateral Nucleus Accumbens***						
Lateral OFC	45,46,47	296	5.12	−53	36	0
DLPFC only	10,9	749	3.99	42	42	16
mPFC	8,10	712	3.45	−16	46	16

*Note*: Results are from region-of-interest analyses focusing on the OFC, mPFC, DLPFC, and ventral striatum. Analyses were thresholded at p_corrected_<0.05 using AlphaSim. *df* = 135. The contrast generated from the reward task was win > loss. Cluster size is in voxels. There were no regions for which (1) alcohol dependent adults exhibited greater response than healthy adults or (2) less negative functional connectivity with the accumbens than healthy adults. There were null findings for positive functional connectivity. OFC: orbitofrontal cortex. mPFC: medial prefrontal cortex. DLPFC: dorsolateral prefrontal cortex.

### Group Differences in Frontostriatal Functional Connectivity

**Figure 3:**
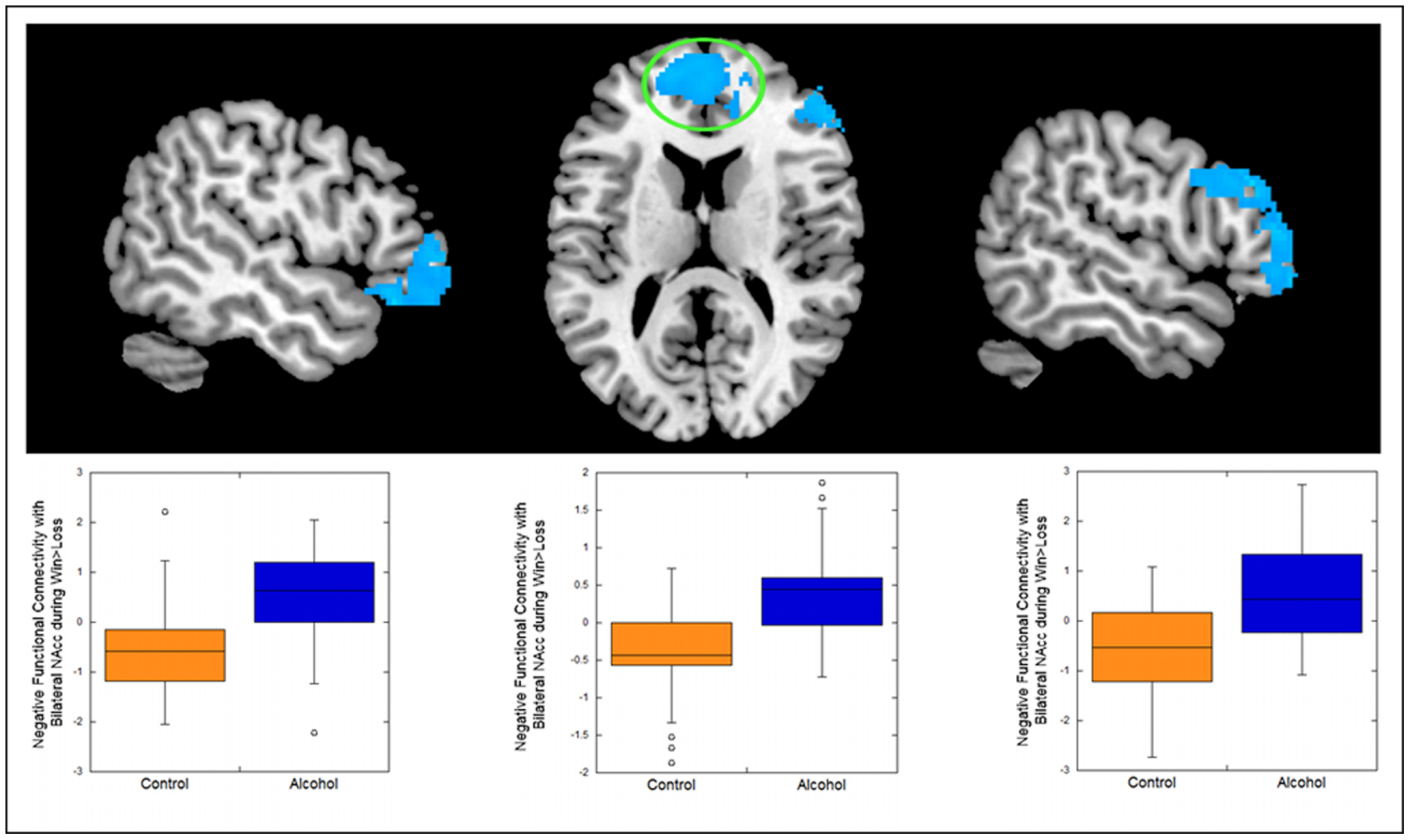
Alcohol dependent adults exhibited stronger negative functional connectivity than healthy adults between the bilateral nucleus accumbens and the lateral orbitofrontal cortex (left), medial prefrontal cortex (center), and the dorsolateral prefrontal cortex (right) during the receipt of monetary reward. Boxplots illustrate findings by depicting mean functional connectivity across the entire indicated functional cluster for each region, by group.

Given the role of prefrontal regions in modulating responding in the VS and the pattern of group differences we observed in response of PFC regions, we focused on functional connectivity between the bilateral nucleus accumbens and the lateral OFC, mPFC, and DLPFC during the experience of winning money compared with losing money. The alcohol dependent group displayed stronger negative functional connectivity than the comparison group—that is, a stronger negative correlation in task-related function— between the nucleus accumbens and all 3 prefrontal ROIs tested (see Table 1; Figure 3). That is, the alcohol dependent group showed stronger associations than the comparison group between nucleus accumbens and prefrontal regions during the context of receiving reward vs. losing. Whole-brain analyses confirmed that these PFC regions exhibited negative functional connectivity with the bilateral accumbens. The groups did not differ for positive functional connectivity. There were no sex differences in functional connectivity.

### Associations between Drinking Characteristics and Brain Function in Alcohol-Dependent Adults

**Figure 4:**
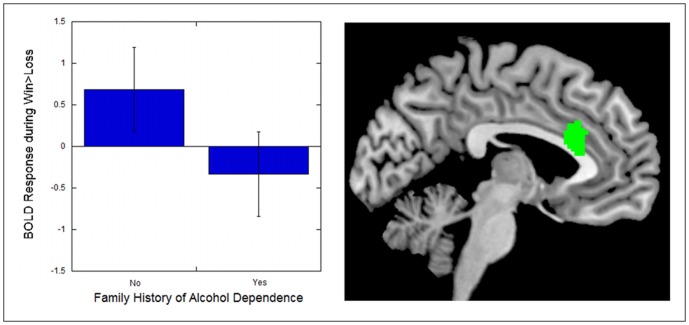
Alcohol dependent adults with a family history of alcohol use disorders exhibited less medial prefrontal response to monetary win vs. loss. The boxplot illustrates findings by depicting mean BOLD response across the entire indicated functional cluster, by family history group.

To investigate the mechanisms of association between alcohol dependence and altered neural response among problem drinkers, we conducted regression analyses with drinking characteristics as regressors. All analyses were conducted within the alcohol dependent group, and they focused on the ROI clusters that emerged from group-difference analyses above. This way, we could examine brain-behavior associations that were relevant to group differences. Age was included as a covariate in these analyses, as it was related to several drinking characteristics (e.g., older participants had spent a greater number of years drinking; *r* = .82 for years drinking, .45 for drinks per use, .45 for frequency of use, .46 for severity, all *p*s<.03). A positive family history of alcohol dependence was associated with low response in anterodorsal cingulate subregion of the mPFC (Table 3; Figure 4).

**Table 3:** Association of Alcohol Dependent Adults' Drinking Characteristics with Function in Reward Circuitry.

Regions	BA	Cluster Size	*t*-score at peak voxel	Talairach coordinates of peak voxel		
				x	y	z
***Family History, Negative Correlation***						
mPFC	24,32	406	3.48	−1	16	29
***Frequency of Use, Negative Functional Connectivity with Bilateral Nucleus Accumbens***						
mPFC	10	257	2.99	−1	46	19

*Note*: Analyses were thresholded at p_corrected_<0.05 using AlphaSim and were constrained using findings for group differences in which the alcohol dependent group exhibited less response than the healthy control group. The contrast generated from the reward task was win > loss. Age was included as a covariate in model of frequency of use. Cluster size is in voxels. mPFC: medial prefrontal cortex.

### Associations between Drinking Characteristics and VS-PFC Functional Connectivity in Alcohol-Dependent Adults

**Figure 5:**
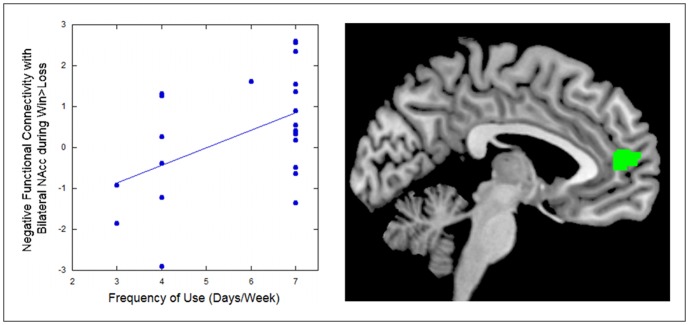
Alcohol dependent adults with more frequent alcohol use exhibited stronger negative connectivity between the bilateral nucleus accumbens and the medial prefrontal cortex and medial orbitofrontal cortex during the receipt of monetary reward. The scatterplot illustrates findings by depicting mean functional connectivity across the entire indicated mPFC functional cluster (y axis) in relation to frequency score (x axis).

As with our analyses of drinking characteristics and neural response to reward above, we conducted regression analyses with drinking characteristics and functional connectivity between the bilateral nucleus accumbens and the regions that distinguished alcohol and control groups' functional connectivity during the experience of winning money compared with losing money. Frequency of drinking (days/week) was associated with greater functional connectivity between the bilateral nucleus accumbens and a cluster including portions of the perigenual anterior cingulate and rostral mPFC (Table 3; Figure 5). That is, alcohol dependent adults who drank alcohol more often exhibited a stronger association between nucleus accumbens and mPFC in the context of receiving a reward vs. losing.

## Discussion

Focusing on the hypothesis that alcohol dependence is accompanied by hypofrontality, we found that young adults with alcohol dependence, compared with healthy young adults, exhibited less response in the lateral OFC, mPFC, and DLPFC in response to monetary reward. In addition, alcohol dependence was associated with a greater negative functional connectivity (i.e., negative correlation in task-related response) between those prefrontal regions and the bilateral nucleus accumbens. Furthermore, suggesting blunted response to non-alcohol reward, young adults with alcohol dependence exhibited less response to monetary reward in the ventral striatum. Relevant to the pathophysiology of alcohol dependence, young adults with alcohol dependence exhibited associations between drinking characteristics and both mPFC response and mPFC-accumbens functional connectivity. Family history of alcohol dependence was related to less mPFC response, and drinking frequency was related to greater negative functional connectivity between the nucleus accumbens and the mPFC. Together, these findings suggest that function in frontostriatal reward circuitry is altered in alcohol dependence, with less response in key PFC regions, disrupted coordination between frontal regions and VS in response to non-alcohol rewards, and associations between drinking characteristics and these frontostriatal alterations. These findings could indicate that alcohol dependence develops through weakened prefrontal regulation of striatal responding, which corresponds to difficulty with behavioral regulation. Because we focused on frontostriatal functional connectivity during winning relative to losing money, our results suggest that altered coordination of VS with mPFC occurs in response to reward rather than to feedback in general. Alternatively, altered coordination between these two reward regions could be weak during rewarding experiences but preserved or strong during loss or punishment experiences. The mechanisms of such weakened prefrontal regulation could include both heritable factors and pattern of exposure to alcohol.

Our findings are consistent with the putative disruptions to components of reward circuitry in addiction. The mPFC was central to our findings, with altered function in response to reward, altered coordination with the nucleus accumbens, and functional associations with pathophysiologic characteristics of alcohol dependence. This region has several key functions in the processing of reward, including both responding directly to reward as a target of midbrain dopamine neurons and serving as a regulatory region for striatal response to reward [8]. The dorsal mPFC is also thought to work in coordination with the DLPFC to regulate affective response [35]. Thus, our findings of less mPFC response to monetary reward in adults with alcohol dependence—and in those alcohol-dependent adults with a family history of the disorder—suggest that low responding in this region could reflect ineffective modulation of basic reward responding in a way that facilitates addictive behavior. This change in modulation could be a stable vulnerability in those with high familial loading for alcohol-related problems. Our findings of altered mPFC functional connectivity with the nucleus accumbens in alcohol dependent adults, as well as altered functional connectivity in more frequent drinkers in the alcohol-dependent group, occurred during the comparison of receiving money and losing money. Thus, striatal-mPFC coordination could be particularly sensitive to rewarding outcomes rather than outcomes generally. These findings have meaning for the function of reward circuitry associated with alcohol dependence specifically and with addiction in general.

The lateral OFC mediates the influence of rewarding experiences on executive function [36] and appears to play a role in higher-level response to reward. Additionally, baseline function in the lateral OFC is associated with positive emotionality, an affective trait related to reward sensitivity and inversely associated with addiction [37]. Although models of OFC function have postulated that lateral OFC is specialized for processing loss rather than reward, a more recent conceptual stance is that lateral OFC also contributes to reward association learning by assigning credit to rewards and re-evaluating rewards [38]. Less response in this area could thus be interpreted as difficulty with updating the value of rewards, which could lead to reduced flexibility and greater compulsivity in responding.

The DLPFC, while not studied as intensively as other prefrontal regions in relation to reward or in populations with alcohol dependence, has emerged as an important region in addiction. Altered DLPFC function in addiction is thought to reflect disrupted decision-making away from flexible responding and in favor of compulsive behavior [6], [39]. In addition, DLPFC is postulated to play a critical role in the self-regulation of reward consumption, for example by modulating the processing of reward value in the service of goals [40], [41], [42]. Alcohol-dependent adults exhibit reduced DLPFC response to cognitive control [43], and those with greater DLPFC response report greater craving [43] and are more likely to seek treatment [44]. Previous studies of alcohol use disorder have indicated that greater negative functional connectivity between the ventral striatum and DLPFC during operant conditioning is associated with slower learning from reward prediction errors and greater craving [45]. While our task involves other aspects of reward processing rather than learning, our findings could suggest a similar alteration of prefrontal influence over reward processing. That is, instead of reduced prefrontal influence on the efficiency of reward learning, as in that previous study, we could have observed reduced prefrontal influence on another process, the initial response to reward. This process is likely to influence a variety of other reward-related functions, including learning.

In addition, altered PFC response to rewarding events has been reported in both adults with substance dependence and adolescents at risk for alcohol use problems. Bjork et al. [46] reported that substance dependent adults exhibited less response to obtaining risky rewards in a posterior dorsal region of mPFC whose function is associated with conflict monitoring and reward-driven behavior. While the mPFC region distinguishing alcohol dependent and comparison groups in the current study is located in a more anterior area of PFC than the region reported in that previous study, our findings suggest a similar pattern of low PFC engagement during reward processing. In our case, and consistent with the nature of our fMRI task, the mPFC subregion with lower function in alcohol dependence is associated with the expression and regulation of response to pleasant stimuli [47].

Our finding of low dorsal and ventral striatal response to monetary reward in adults with alcohol dependence is consistent with other findings in alcohol dependence, even those focusing on the anticipation of reward rather than rewarding outcomes [20], [21]. In the context of the extant literature on response to drug vs. non-drug reward in alcohol dependence, the current findings for VS response provide partial support for the hypothesis that addiction shifts reward function away from natural or non-drug rewards—in this case, money—and toward drug rewards [48]. Notably, the accompanying hypothesis that alcohol dependence is associated with altered frontostriatal functional connectivity during reward processing is not inconsistent with low VS response in alcohol dependence. Low response within the VS does not guarantee or preclude altered coordination with frontal regions during the experience of a task. To address this issue in greater depth, research designs that include both drug and non-drug rewards in a single fMRI paradigm will therefore be valuable in future studies. While our findings contribute to what is known about neural response to non-drug rewards in addiction, a more powerful examination of differences in response to drug rewards and non-drug rewards would be provided by fMRI paradigms that incorporate both types of reward.

Notably, we did not find that alcohol dependent adults differed from healthy adults in the response or functional connectivity of the medial OFC. Given the putative role of medial OFC in representing reward value [49], it is possible that our paradigm, with its fixed reward values, did not elicit meaningful differences related to alcohol dependence. The presence of findings for lateral but not medial OFC supports the value of considering these subregions of OFC separately. In addition, our findings for mPFC underscore the importance of considering the medial OFC (or ventral mPFC) distinct from the mPFC, as the two regions differ functionally and have distinct patterns of connectivity with the striatum [8]. Alternatively, alcohol dependence might be more strongly associated with altered function in prefrontal regions with a role in regulating or promoting flexibility in behavior.

Our analyses with drinking characteristics allowed us to elucidate some of the potential mechanisms of group differences between alcohol-dependent and healthy adults. Although we were limited somewhat by not having collected similar data from healthy participants, we were able to investigate the altered frontostriatal function within the alcohol dependent group in greater depth. Our study design did not focus on relapse or on the association of abstinence symptoms with frontostriatal function, but detailed examination of these issues will be worthwhile for future studies. In addition, the individual differences associated with the likelihood and timing of relapse could be a fruitful topic for investigations of the pathophysiology and course of alcohol dependence.

The current sample was somewhat unusual for a study of alcohol dependence because participants were young and high functioning, had no Axis I disorders, and were not seeking treatment. These characteristics allowed us to focus on the function in frontal regions that are components of dopamine systems with minimal confounding by ageing or long-term alcohol exposure. In addition, these sample characteristics allow us to interpret our findings in light of the literature on frontostriatal function in young people at risk for alcohol dependence. We acknowledge that in many ways, the current study is more an examination of the pathophysiology of alcohol dependence than an examination of its development. Ideally, the field needs prospective studies that include the developmental window allowing assessment of the initiation of alcohol use and development of alcohol dependence. Relevant to this need, findings from the large, multisite IMAGEN consortium have contributed importantly to our understanding of the role of reward circuitry in young people at risk for addiction or early in the process of substance use (e.g., [50], [51], [52]). This study has reported that adolescents who are smokers [50], had prenatal exposure to nicotine [51], or had “potentially problematic” substance use [52] exhibit low VS responsiveness to reward. Similarly, although we were not able to examine brain function before and after onset of alcohol use or dependence, we note that our sample has unique features that allow us to investigate frontostriatal function early in the course of alcohol use problems.

Recent developmental findings have begun to elucidate the role of reward circuitry in the process of addiction. For instance, adolescents at high familial risk for alcohol use problems exhibit low DLPFC response during risky decision-making [53] and less VS response to reward [26], suggesting that altered prefrontal responding to reward could be an endophenotype present in those who are vulnerable to alcohol use problems before they have used alcohol. However, recent findings of the IMAGEN study indicate that the total contribution of response to reward in a set of reward-related neural regions (including PFC and VS) contributes only modestly to the initiation of alcohol use in early adolescence [54]. The authors of this study interpreted their findings as indicating that the function of reward circuitry contributes more importantly to the processes underlying the development of alcohol dependence than to those underlying the initiation of alcohol use. In distinction to our findings on altered frontostriatal connectivity, young adults at risk for alcohol dependence have been reported to exhibit altered functional connectivity between the accumbens and non-PFC regions such as the precuneus and supplementary sensorimotor area [55]. Together, these findings suggest that response in reward-related neural regions could differ for those with a family history of alcohol dependence but the absence of current problems. In contrast, rather than being a trait-like characteristic or precursor, altered functional connectivity between PFC and accumbens might instead emerge with the process of addiction.

Current and past findings cannot definitively settle the meaning of our findings of altered PFC function and frontostriatal connectivity, but they provide a context for understanding these findings. Without a prospective, longitudinal design, it is impossible to separate the role of frontrostriatal function as a potential influence on the development of alcohol dependence from its function as a consequence or correlate of alcohol dependence. Indeed, our sample of alcohol-dependent adults, while relatively early in development and relatively young among alcohol-dependent adults, had spent an average of 10 years drinking. This exposure to alcohol is likely to have influenced function in frontostriatal circuitry, especially given its developmental timing. Notably, exposure to alcohol can have particularly pernicious effects on brain development [56], [57], and our sample may be valuable for studying the consequences of early-onset alcohol-use problems. Furthermore, given the changes of function in reward circuitry during adolescent development [34], [58], [59], studies must also account for developmental processes as well as alcohol exposure and addiction as longitudinal influences on reward circuitry. More specifically, prospective studies should examine changes in VS response (i.e., possible decreases in sensitivity to non-drug reward stimuli over time), PFC response (i.e., stably low or decreasing level of response to reward), and frontostriatal connectivity (i.e., negative correlation between VS response and PFC response during reward processing).

Several other factors related to our sample composition constrain our interpretations. We were not able to examine sex differences, as our sampling strategy was designed to minimize differences by matching the alcohol dependent and healthy control groups for this and other demographic characteristics. We did not measure cognitive ability (e.g., IQ) or match groups on it, but this characteristic could have contributed to function in the circuits of interest in the current study. In addition, we did not collect detailed information about family history of alcohol dependence beyond its presence or absence of a first- or second-degree relative. As a result, we were not able to investigate the effects of family history density (e.g., as applied in work by Stoltenberg et al. [60] and Zucker et al. [61]) or of alcohol dependence in particular family members (e.g., mothers) on neural response or functional connectivity. Finally, we did not collect data on alcohol use from participants in our healthy control group, and this would have allowed us to draw stronger conclusions about the mechanisms of group differences in frontostriatal function.

In sum, our findings provide evidence for hypofrontality and altered frontostriatal connectivity in young adults with alcohol dependence. Low responding in these regions, which have roles in self-regulation and higher cognitive function, likely contributes to the compulsive pursuit of alcohol, the poor behavioral flexibility, and the loss of control that accompany this disorder. In addition, altered functional connectivity between prefrontal regions and the VS indicates that coordination within reward circuitry is disrupted in alcohol dependence. Placed in the context of the literature on risk for alcohol dependence, these findings raise the hypothesis that weakening of frontal modulation of VS occurs early in the development of alcohol dependence.

## Supporting Information

Table S1 **Results of Whole-Brain Analyses Testing Differences between Alcohol-Dependent and Healthy Young Adults in Neural Response and Functional Connectivity with the Bilateral Nucleus Accumbens during Reward Outcome.**
(DOCX)
