# Supplementary material for: Prefrontal Response and Frontostriatal Functional Connectivity to Monetary Reward in Abstinent Alcohol-Dependent Young Adults
Source: PLoS One. 2014 May 7;9(5):e94640. doi: 10.1371/journal.pone.0094640 (PMC4012954; doi:10.1371/journal.pone.0094640)
Supplement: Table S1 — Results of Whole-Brain Analyses Testing Differences between Alcohol-Dependent and Healthy Young Adults in Neural Response and Functional Connectivity with the Bilateral Nucleus Accumbens during Reward Outcome. (DOCX) [file pone.0094640.s001.docx]

**Table S1.** Results of Whole-Brain Analyses Testing Differences between Alcohol-Dependent and Healthy Young Adults in Neural Response and Functional Connectivity with the Bilateral Nucleus Accumbens during Reward Outcome

| Regions in Cluster | BA | Cluster Size | *t*-score at  peak voxel | Talairach coordinates of peak voxel | | |
| --- | --- | --- | --- | --- | --- | --- |
|  |  |  |  | x | y | z |
| ***Alcohol Dependent < Healthy Control, Neural Response*** | | | | | | |
| Lateral OFC, Insula | 13, 47 | 565 | 5.69 | 32 | 12 | 1 |
| mPFC | 32 | 2615 | 5.49 | 2 | 27 | 32 |
| DLPFC | 8,9,6 | 608 | 5.40 | 39 | 19 | 43 |
| Lateral OFC | 10,11 | 332 | 5.05 | 33 | 51 | 2 |
| Inferior Parietal Lobule | 40 | 490 | 5.05 | 50 | -52 | 36 |
| Insula | 13 | 474 | 5.05 | -36 | 10 | -1 |
| DLPFC, Lateral OFC, Temporal Pole | 45,46,38, 47 | 100 | 4.38 | -58 | 31 | 1 |
| mPFC | 23,31 | 210 | 4.32 | 4 | -21 | 28 |
| DLPFC | 45,42,44 | 138 | 4.30 | -62 | 12 | 19 |
| DLPFC | 8,9 | 136 | 4.11 | 19 | 48 | 33 |
| Dorsal Striatum, VS |  | 183 | 4.04 | 12 | 1 | 14 |
| Lateral OFC | 10 | 60 | 3.99 | -23 | 50 | -6 |
| DLPFC | 9,10 | 141 | 3.86 | -23 | 47 | 28 |
| ***Alcohol > Control for Negative Functional Connectivity with Bilateral Nucleus Accumbens*** | | | | | | |
| mPFC | 8,6 | 72 | 5.25 | 0 | 38 | 55 |
| Lateral PFC, mPFC, Medial OFC, DLPFC | 10,11,46 | 532 | 4.40 | 32 | 48 | 9 |
| Caudate Body |  | 234 | 4.39 | 10 | 18 | 15 |
| Visual Cortex, Cuneus | 17,18 | 116 | 4.37 | 14 | -98 | -8 |
| Brainstem (Pons) |  | 75 | 4.08 | 12 | -18 | -28 |
| DLPFC | 9 | 72 | 3.79 | 41 | 33 | 32 |

*Note*: The contrast generated from the reward task was win > loss. Cluster size is presented in voxels. The first region or Brodmann Area is the location of the peak voxel. Analyses were thresholded at *p* < .001 and extent = 50 voxels. OFC: orbitofrontal cortex. mPFC: medial prefrontal cortex. DLPFC: dorsolateral prefrontal cortex. VS: ventral striatum.
